# Supplementary material for: Patterns of Intron Gain and Loss in Fungi
Source: PLoS Biol. 2004 Nov 30;2(12):e422. doi: 10.1371/journal.pbio.0020422 (PMC532390; doi:10.1371/journal.pbio.0020422)
Supplement: Table S1 — Also available at http://genes.mit.edu/NielsenEtAl/. (4.3 MB ZIP). [file pbio.0020422.st001.zip › NielsenEtAl/html/262.html]

AN9504.1.NCU03187.1.MG03026.1.FG09408.1


```
 CLUSTAL W (1.82) Multiple Sequence Alignments - Introns Inserted


Sequence 1: NCU03187.1	822 aa
Sequence 2: FG09408.1	715 aa
Sequence 3: MG03026.1	591 aa
Sequence 4: AN9504.1	699 aa
Alignment Length: 837 aa
Number Identitical Residues: 237 aa
Alignment Score (without introns) 12087


MG03026.1 	----MPESDNYEVLEKIG1HGSFGVIRKVRRKSDGM~VVCRKEISYLKMSQKEREQLHAE
NCU03187.1	----MSESDKYELLEKIG1HGSFGIIRKVRRKADGM~ILCRKEISYLKMSQKEREQLHAE
FG09408.1 	----MSSEDKYETLEKIG1HGSFGIIRKVRRRTDGF~IMCRKEISYLRMSQKEREQLHAE
AN9504.1  	MAIALAEADKYEVLEKIG1CGSFGIIRKVKRKSDGF0ILCRKEINYIKMSTKEREQLTAE
          	 : ::.. *:** *****  ****:****:*::**: ::*****.*::** ****** **

MG03026.1 	FQILSTLHHENIVGYFHREHLKSSQDLYLYMEYCGNGDLGRVIRDLQTKNQYAEESFVWS
NCU03187.1	FSILSTLRHPNIVGYYHREHLKATQDLHLYMEYCGNGDLGRVIRNLIKNNQYAEESFVWS
FG09408.1 	FQILSHLRHPNIVAYYHREHLKVSQDLHLYMEYCGNGDLGRVIKDLALKGQRAQESFVWS
AN9504.1  	FNILSSLRHPNIVAYYHREHLKASQDLYLYMEYCGGGDLSMVIKNLKRTNKYAEEDFVWR
          	*.*** *:* ***.*:****** :***:*******.***. **::*  ..: *:*.*** 

MG03026.1 	IFSQLVTALYRCHYGINPPEVGKSLFGPVK---STKPVIPPGTMTILHRDLKPENV1FLG
NCU03187.1	IFSQLVTALYRCHYGVDPPEVGKTVLGLGS---TARPKPPSGGMTILHRDLKPENV1FLG
FG09408.1 	IFSQLVMALYRCHYGVDPPEVGSNILGLTQGNAAAGPKVPAGTMTILHRDLKPENV1FLG
AN9504.1  	ILSQLVTALYRCHYGTDPAEVGSNLLGPAP--KPSGLKGKQAQMTILHRDLKPENI~FLG
          	*:**** ******** :*.***..::*      .:      . ************: ***

MG03026.1 	EGNLVKLGDFGLAKMIQSQDFASTYVGTPFYMSPEICAAEKYTLKSDIWSLGCIIYELCT
NCU03187.1	EDNSVKLGDFGLSKVMQSHDFASTYVGTPFYMSPEICAAEKYTLKSDIWSLGCIIYELCA
FG09408.1 	EDNSVKLGDFGLSKMIKSHDFASTYVGTPFYMSPEICAAEKYTLKSDIWSLGCIIYELCA
AN9504.1  	SDNTVKLGDFGLSKLMHSHDFASTYVGTPFYMSPEICAAEKYTLRSDIWAVGCIMYELCQ
          	..* ********:*:::*:*************************:****::***:**** 

MG03026.1 	REPPFNAKTHVQLVQRIREGKISPIPKHYSPDLMEVIKECLRVNPERRPDTATLLHLPHV
NCU03187.1	REPPFNAKTHYQLVQKIKEGKIAPLPSVYSGELFATIKDCLRVNPDRRPDTATLLNLPIV
FG09408.1 	REPPFNAKTHFQLVQKIKDGKFPALPDVYSPELYQVIKDCLRVNPDRRPDTSELLNLPVV
AN9504.1  	REPPFNARTHIQLVQKIREGKFAPLPDFYSSELKNVIASCLRVNPDHRPDTATLINTPVI
          	*******:** ****:*::**:..:*. ** :*  .* .******::****: *:: * :

MG03026.1 	KMMRKEKELVDCNRMLKQKQE---------------------AIRKELNASLHREWEVKA
NCU03187.1	RLMRKEKEVVEFSRTLRTKEETLNKRIRELDSKLSALETEKSSIRAEIDASLRREWEVKA
FG09408.1 	KLMRKEKEVVDLNKSIRLREDSLRKKERDLNERLVNMEREKELIRDELDSSLRREWEVKA
AN9504.1  	RLMRREVELNNLSRAARKREEATMQKAKDVEQAFAKLEKEKQQIRSELENSIRREWEVKA
          	::**:* *: : .:  : ::::  .   . ..     . ... ** *:: *::*******

MG03026.1 	RLEIDRILKEEIAGLEKRFEAEVQKRIEEDRQKR-----------------PEMDYQKRF
NCU03187.1	RLEIDRLVAQEIESLQQKFEQEVQARVEAELQRHGRGPMFNSHGQQGSFSSTAATLVSDY
FG09408.1 	RLEIDRLANAEIEQLQNRFEEEVQARVEAELQKK-AAAYANSRPNSQEDEYTTSTGKTDY
AN9504.1  	RLEIDRQVQNELDKLRKRFECEVQDRVAQEVEKQ-------RRNANYREDASLRSSGHSS
          	******    *:  *.::** *** *:  : :::           .   . .        

MG03026.1 	EMDYQKRFEADYQKRFDLEFPKKLE-EEMR----RRYPDTP----------------GSK
NCU03187.1	NLSSVGSGDGDFPSTTDITDISIAE-STDGSDITKKIPRTPFHRAQTYSSAPAESVLGTP
FG09408.1 	PHSSVGGSEVEFPSTTDLTEYSSIE-SPEAPRETKKTARTPFGRAQTMFVG---NPAGTP
AN9504.1  	QMSSSNSEDSDFPSSTDISQLSLESPTNKAAKLPKKESRTPFTRSKT--------VVDSP
          	  .     : :: .  *:   .  ..    .  .:: . **   :.:          .: 

MG03026.1 	MDLSWSVPKPKASKSQSSVEEVSADG---DESSGPDQPDLSGTSPDSSFSNSNHNEP---
NCU03187.1	MDIEMASPSPITIASLSLSPRRMAL----TKAPTTNPRMIFGEEP-TSTDKSNWEVPRET
FG09408.1 	MDIEMNSPSPIAIASLSLSPRRNGN----TKAPTAASGNIFAVNANRSADNSRWDLR---
AN9504.1  	MDIQMAEPSPISIASLSLSPRRTSATYSGKNIFAEGERKRPKFEPTLAYSDDEDDTP---
          	**:.   *.* :  * *   .  .   :. :            ..  : .... :     

MG03026.1 	---------------------KRRPRTPY----KRAQTMYAGGGRAAGTPMDIEMVSPSP
NCU03187.1	EMIDSGDESEAEALVPSPKRITKSSKNPFSTVTTRSRPSLNSQQNSNVLPIHGLRSK-QT
FG09408.1 	---DTLSDSEDEDVMPSPTRNIKSSKNPF---TSKTRPVLTSQKS---APINRLKSQPST
AN9504.1  	-------------ELPSPTRPKVKP-DPFK---APSRPLLRQNTTALMQKLSTQPPIFPA
          	               .:..     .  *:.     ::.       :    :        .

MG03026.1 	IAIASLSLS-----------------------------PRRHAATTGSPTKN--------
NCU03187.1	LATRSKTVSGVSSIGQHPLRSAPSAPSLRDRKPSPTRRLSRIPSVTGVGRRLSANNINNS
FG09408.1 	SGFVSKQVS------------QPEAP----RSP---RRLSKIPSAANLQAES-----NNS
AN9504.1  	NPSRLPQMS------------APDVRESKSRSP--HRRLSKIPSSANLAADAG-------
          	       :*             ..  .  . ..       : .: :.     .       

MG03026.1 	---------------------------------------------------------PFS
NCU03187.1	SNGGSDAPSSTVTSNITVRTRGLKRMSSTCDESSFSQQQNNQPQQSLPQAPPLKKIGLMA
FG09408.1 	SGGG------------LTRQSSLNRKNNHSDDG----------------------LGKVA
AN9504.1  	---------------SPTRKNGVKSSPSKMNGGD--------------------EMFKAV
          	                     . .   .  . ..                    .     

MG03026.1 	KRNNVQGRTLVELSKARASGRPL-----------------------FDKSNLISRS---E
NCU03187.1	AKN-IRGSSLVELHQARAGGRPISAIISNEAKLRAFKEHATIAASAVDSSSSSSSSSGQS
FG09408.1 	AKNNIRGRTLVELQQARAGGRPMSAIV-----MPSTGENVSPKRAFRDRIGAERKSSGDE
AN9504.1  	MQRNMGGRTLVELAQARAGGRPIDEVKR-------------CASDSRSGCSVPMKSADRD
          	 :..: * :**** :***.***:.                       .  .    *:. .

MG03026.1 	PVATWNPEIDEMPSPFLVRRKGVMPP~VSAGR----------------------------
NCU03187.1	QLPTRPRSQPQPITANFEQQQQQQQI1NAMAGSSRMESGGGDGGNAQSVYCEDLEKAGEL
FG09408.1 	PVAVWDPERDEMPSPFLVRQRRIARV~---------------------------------
AN9504.1  	PPAVWDPERDEMPSPFLARGRKVIRN~LR-------------------------------
          	  ..   .  :  :. : : :                                       

MG03026.1 	-
NCU03187.1	C
FG09408.1 	-
AN9504.1  	-
          	
```
